# Supplementary material for: Magnon thermal Hall effect via emergent SU(3) flux on the antiferromagnetic skyrmion lattice
Source: Nat Commun. 2024 Jan 23;15:566. doi: 10.1038/s41467-024-44793-3 (PMC10805809; doi:10.1038/s41467-024-44793-3)
Supplement: Supplementary file 1 — Supplementary Information [file 41467_2024_44793_MOESM1_ESM.pdf]

Supplementary Information

Hikaru Takeda,[*] Masataka Kawano,[†] Kyo Tamura, Masatoshi Akazawa, Jian Yan, Takeshi Waki, Hiroyuki Nakamura, Kazuki Sato, Yasuo Narumi, Masayuki Hagiwara, Minoru Yamashita, and Chisa Hotta

I. SUPPLEMENTARY NOTE 1: EXPERIMENTAL

A. Detailed setup of the thermal transport measurements

As shown in Supplementary Figs. 1(a) and 1(b), one heater and three thermometers ($T_{\rm High}$, $T_{\rm L1}$, and $T_{\rm L2}$) were attached to the sample using a silver paste. The sample size is about $1.0 \times 0.8 \times 0.07$ mm^3. To avoid a background signal coming from metal, the sample was attached to the insulating LiF heat bath with non-metallic grease. The heat current $J_{\rm Q}$ and the magnetic field B were applied along [111] and [321] directions of the sample, respectively. Both the longitudinal $\Delta T_x (\Delta T_x = T_{\rm High} - T_{\rm L1})$ and the transverse $\Delta T_y (\Delta T_y = T_{\rm L1} - T_{\rm L2})$ temperature differences were measured as a function of the heat current $J_Q = Q/wt$, where Q is the heater power, t is the thickness of the sample, and w is the mean sample width.

Supplementary Figure 2 shows a representative time profile of the sample temperature ($[T_{\rm High} + T_{\rm L1}]/2$), ΔT_x and ΔT_y measured at 0.5 K and 6.5 T. A stable temperature gradient (ΔT_x was typically set about 3–5% of the sample temperature) was well established during the measurement despite the small size of the sample. After waiting for the stabilization of the sample temperature, we averaged the data for 240–300 secs for each measurement. The stability of the sample temperature during the period is typically 0.002% of the sample temperature, which is small enough to resolve $\Delta T_y^{\rm asym}$ of our measurements.

The heater and the thermometers used in our thermal-transport measurements were thermally well isolated from the LiF heat bath by a thin Kapton tube and resistive manganin wires. The thermal resistance between the thermometers and the heat bath is at least two orders magnitude larger than that of the sample in the whole temperature range of our study.

To cancel the longitudinal component in ΔT_y by the misalignment effect, ΔT_y was asymmetrized with respect to the field direction as $\Delta T_y^{\rm asym} = [\Delta T_y(+B) - \Delta T_y(-B)]/2$. To take into account a magnetic hysteresis effect, this antisymmetrization was done separately for the data obtained in the field-up process and that in the field-down process. The thermal (Hall) conductivity $\kappa_{xx}(\kappa_{xy})$ is derived by

$$\begin{pmatrix} Q/wt \\ 0 \end{pmatrix} = \begin{pmatrix} \kappa_{xx} & \kappa_{xy} \\ -\kappa_{xy} & \kappa_{xx} \end{pmatrix} \begin{pmatrix} \Delta T_x/L \\ \Delta T_y^{\rm asym}/w' \end{pmatrix}, \quad (1)$$

where L is the length between the thermal contacts for reading $T_{\rm High}$ and $T_{\rm L1}$ and w' is the length between the thermal contacts for $T_{\rm L1}$ and $T_{\rm L2}$. The irregular shape of the sample and the finite size of the thermal contacts

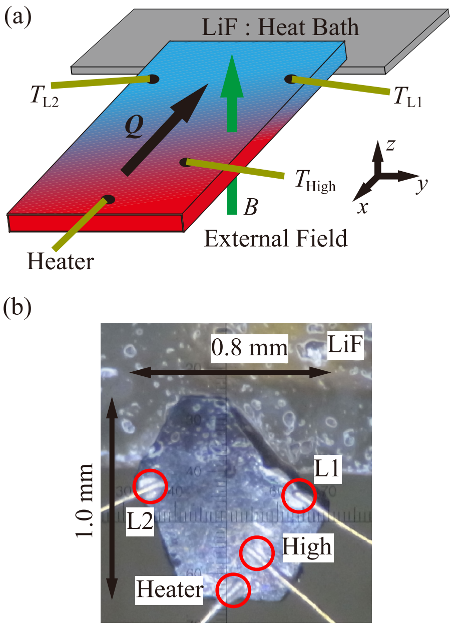

Supplementary Figure 1. (a) Schematic figure of the setup of the thermal transport measurements. (b) Photograph of the sample mounted on the heat bath of LiF. The thermal contacts for the heater and the thermometers are marked by red circles.

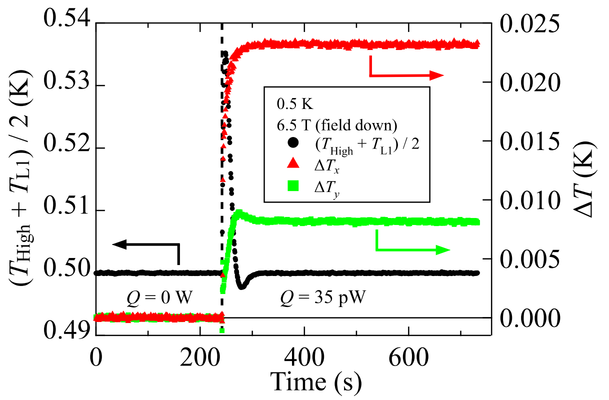

Supplementary Figure 2. Representative time profile of the sample temperature ($(T_{\rm High} + T_{\rm L1})/2$, left axis), $\Delta T_x \equiv T_{\rm High} - T_{\rm L1}$, and $\Delta T_y \equiv T_{\rm L1} - T_{\rm L2}$ (right axis) at 0.5 K and 6.5 T. A heat current $Q = 35$ pW was turned on at the time shown by a dashed line.

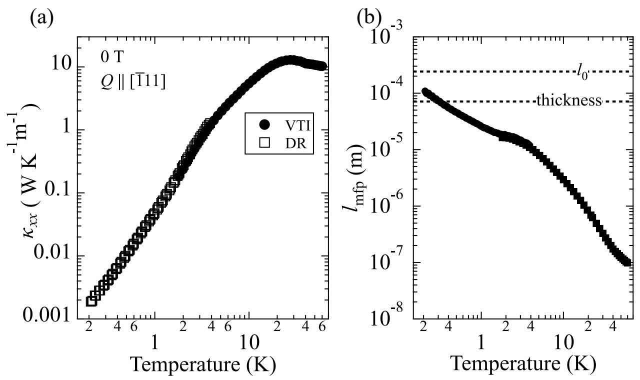

Supplementary Figure 3. (a) Temperature dependence of κ_{xx} at zero external field measured in the variable-temperature insert (VTI, circles) and the dilution refrigerator (DR, squares). (b) Phonon mean free path estimated by assuming that the data in panel (a) is solely from phonons.

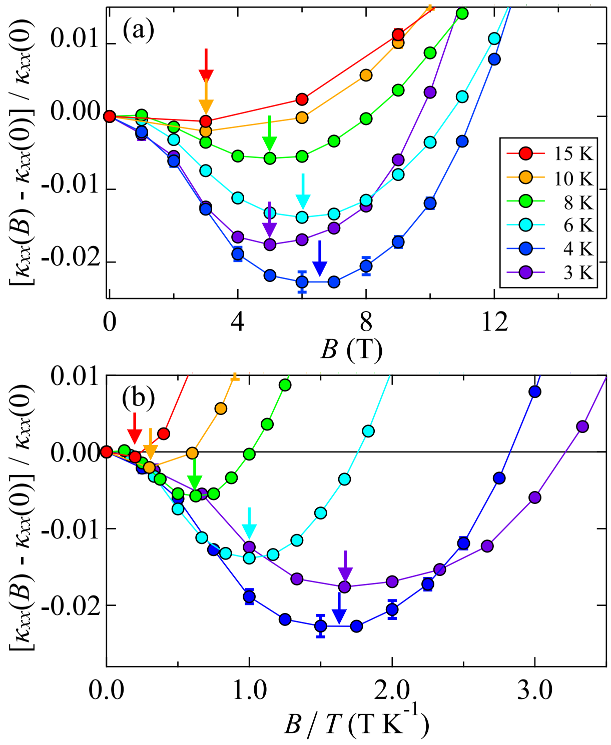

Supplementary Figure 4. Normalized κ_{xx} as a function of (a) B and (b) B/T for several choices of temperatures above T_{N}. Arrows denote the minimum position of normalized κ_{xx}. All the data points are averaged over the field-up and field-down measurements, and the error bars are the maximum deviations of the data from the averages.

cause uncertainty in estimating these geometrical factors, resulting in the ambiguity of the absolute values of κ_{xx} and κ_{xy} by a factor of 2–4.

B. Estimation of the phonon mean free path

Supplementary Figure 3(a) shows the temperature dependence of κ_{xx} in a zero external magnetic field. It exhibits a T^2 behavior without a clear anomaly at the Néel temperature ($T_{\mathrm{N}} = 2.3$ K). Here, we estimate the phonon mean free path (ℓ_{mfp}) by assuming that κ_{xx} is only given by phonons ($\kappa_{xx}^{\mathrm{ph}}$) as shown in Supplementary Fig. 3(b).

The process of evaluating ℓ_{mfp} is given as follows. The thermal conductivity of phonons is given by

$$\kappa_{xx}^{\mathrm{ph}} = \frac{1}{3} C_{\mathrm{ph}} v_{\mathrm{ph}} l_{\mathrm{ph}}, \tag{2}$$

where C_{ph}, v_{ph}, and l_{ph} are the heat capacity, the sound velocity, and the mean free path of phonons, respectively. Referring to Ref.[1], we obtain the temperature dependence of C_{ph} for acoustic phonons. The sound velocity is estimated as approximately 1770 m/s from the Debye temperature of 154 K.

C. Magnetic field dependence of κ_{xx}

Supplementary Figure 4(a) shows B-dependence of $\kappa_{xx}(B)$ normalized as $(\kappa_{xx}(B) - \kappa_{xx}(0))/\kappa_{xx}(0)$ in the temperature range of $3\,\mathrm{K} \leq T \leq 15\,\mathrm{K}$. The field at which the normalized $\kappa_{xx}(B)$ takes the minimum shifts to higher fields when lowering the temperature from 15 K to 4 K, and then slightly shifts to the lower field at 3 K. The suppression of κ_{xx} by the magnetic field (negative magneto thermal conductivity) can be attributed to the decrease of either κ_{ph} or κ_{mag}.

The former suppression originates from the resonance scattering effect of phonons due to spins, and should be enhanced the most when the energy scale $4k_{\mathrm{B}}T$ that gives the maximum of the density distribution of phonons becomes equal to the Zeeman splitting, $gS\mu_{\mathrm{B}}B$. We plotted the normalized κ_{xx} as a function of B/T in Supplementary Fig. 4(b). If this scenario applies, κ_{xx} should take the minimum at $B/T \sim 4k_{\mathrm{B}}/gS\mu_{\mathrm{B}}$, independent of T, which does not happen here. Therefore, we conclude that the suppression of $\kappa_{xx}^{\mathrm{ph}}$ by the magnetic field is not ascribed to the resonant scattering effect of phonons.

The remaining possibility is the decrease of $\kappa_{xx}^{\mathrm{mag}}$. Since the Weiss temperature is -23 K, the antiferromagnetic correlation will develop at temperatures below 20 K. In fact, the magnetic specific heat is found to increase with decreasing temperature at $T < 15$ K. In $MnSc_2S_4$, in a paramagnetic phase at low temperatures above T_N the magnetic diffuse scattering experiments show the existence of some sort of magnetic excitations relevant to a manifold of wave numbers forming surfaces in the reciprocal space[2]. This clear magnetic feature indicating a correlated paramagnet has a room to couple to magnetic fields and we speculate it to be the origin of the negative field dependence of $\kappa_{xx}^{\mathrm{mag}}$. While this scenario is out

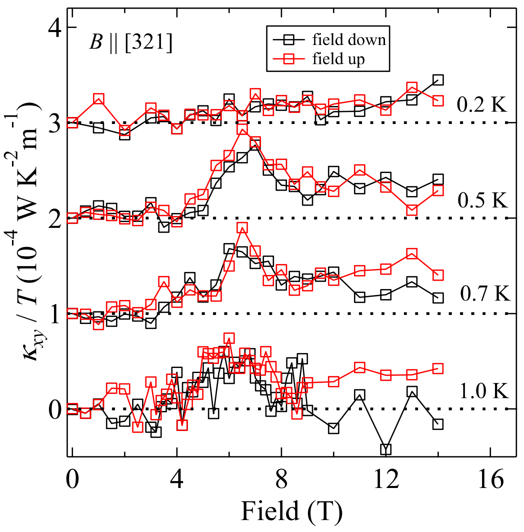

Supplementary Figure 5. Magnetic field B dependence of κ_{xy}/T obtained in the field up and down processes for sample #1.

of scope of the present theoretical treatment, it is consistent with the observed energy scales: the order of the magnetic interaction ∼20 K and the location of minimum ∼ 6 T.

D. Field-up and down processes of κ_{xy}/T

Magnetic skyrmions are only thermodynamically stable (often not in the ground state) and cannot appear without the aid of a magnetic field or strong magnetic anisotropy, and appears often only at finite temperature as found in the previous studies[3–8]. To confirm that the present data measured in a non-equillibrium is stable and reproducible, we measured the thermal Hall conductivity for the field-up and field-down processes. Supplementary Figure 5 shows κ_{xy}/T as a function of field-up and field-down for temperatures $T = 0.2, 0.5, 0.7,$ and 1 K. We find that the two processes agree well, indicating that the phase boundaries do not depend on the processes. This behavior is in sharp contrast to the other skyrmion materials, e.g. GaV_4Se_8, that exhibit different behavior between field-up and down processes. The data points given in Fig. 1(e) in the main text are the ones averaged for the two processes, and the error bars are the maximum deviation of the measured data from the average values.

E. Thermal transport properties in $\boldsymbol{B} \parallel [111]$ investigated by sample #2

The B-T phase diagram of $MnSc_2S_4$ are studied in detail for $\boldsymbol{B} \parallel [111]$ and $\boldsymbol{B} \parallel [110]$[2,9]. Since for the former case, the phase boundary has a simple structure as a function of field, we studied sample #2 which has [111] plane at the sample surface. Here, we compare the results of sample #2 with those of sample #1 shown in the main text.

Supplementary Figure 6(a) shows the temperature dependence of $\kappa_{xx}(B=0)$ in a zero field. Since the quality of sample #2 is lower than that of sample #1, the absolute value is smaller, while they show good agreement in their temperature dependences.

In Supplementary Figs. 6(b)-(d) we plot the field induced components of κ_{xx} and κ_{xy} in three different ways; the same data for the normalized $\Delta\kappa_{xx}(B)/\kappa_{xx}(0)$ is given in panel (b) and the unnormalized $\Delta\kappa_{xx}(B) = \kappa_{xx}(B) - \kappa_{xx}(0)$ is in panel (d). For κ_{xy}/T in panel (c), we basically consider it as a field-induced component because $\kappa_{xy}(0) \sim 0$. We first compare the results of κ_{xy}/T and $\Delta\kappa_{xx}(B) = \kappa_{xx}(B) - \kappa_{xx}(0)$ in Supplementary Figs. 6(c) and 6(d), and find that in both cases, the data of sample #1 is about 10 times larger than that of #2, and their field-dependent functional form agrees very well up to about 9T, which is the range considered to lie in the AFM-SkL phase. These results indicate that the fraction of the field-induced component in sample #2 is about 1/10 of that of sample #1, and their field dependence is very accurately evaluated.

We then find that $\Delta\kappa_{xx}(B)/\kappa_{xx}(0)$, a normalized value by the zero-field one, in Supplementary Fig. 6(b) has about 3.2 larger value for sample #1 than sample #2. This is ascribed to 3 ∼ (10/3.2) times larger value of $\kappa_{xx}(0)$ for sample #1 than sample #2, that appears in the denominator of the data in panel (b). Indeed, $\kappa_{xx}(0)$ of the two samples shown in Supplementary Fig. 6(a) have the same temperature dependent slope in the log-log plot, and differ only by their amplitude that agrees with the constant scaling factor ∼ 3 throughout the temperature range we measured.

Here, $\kappa_{xx}(B \geq 0)$ consists of contributions from phonons and magnons, and although the two contributions is not clearly separable, it is natural to expect that the magnons is responsible for a major part of the field dependent component, $\Delta\kappa_{xx}(B)$. On the other hand, in a zero field, the phonons have large contributions, and the factor three difference of $\kappa_{xx}(0)$ of the two samples in Supplementary Fig. 6(a) indicate a large difference in the extrinsic effect and shall be phenomenologically ascribed to the difference in the phonon mean-free path or to the lifetime.

Basically, the zero field value $\kappa_{xx}(0)$, and the field dependent $\Delta\kappa_{xx}(B)$ or $\kappa_{xy}(B)$ have no rigid reason to be related. However, experimentally, in various magnetic insulators, the sample with smaller amplitude of $\kappa_{xx}(0)$ is found to have smaller $\kappa_{xy}(B)$[10]. The annomalous Hall effect of electrons in ferromagnetic metals, σ_{xy} originating from the Berry curvature effect is considered to be independent of σ_{xx}, but in reality it is not completely free from the scattering effect. The scattering broadens the conduction bands and suppress the intrinsic σ_{xy} as well as σ_{xx}[11]. These facts are consistent with the over-

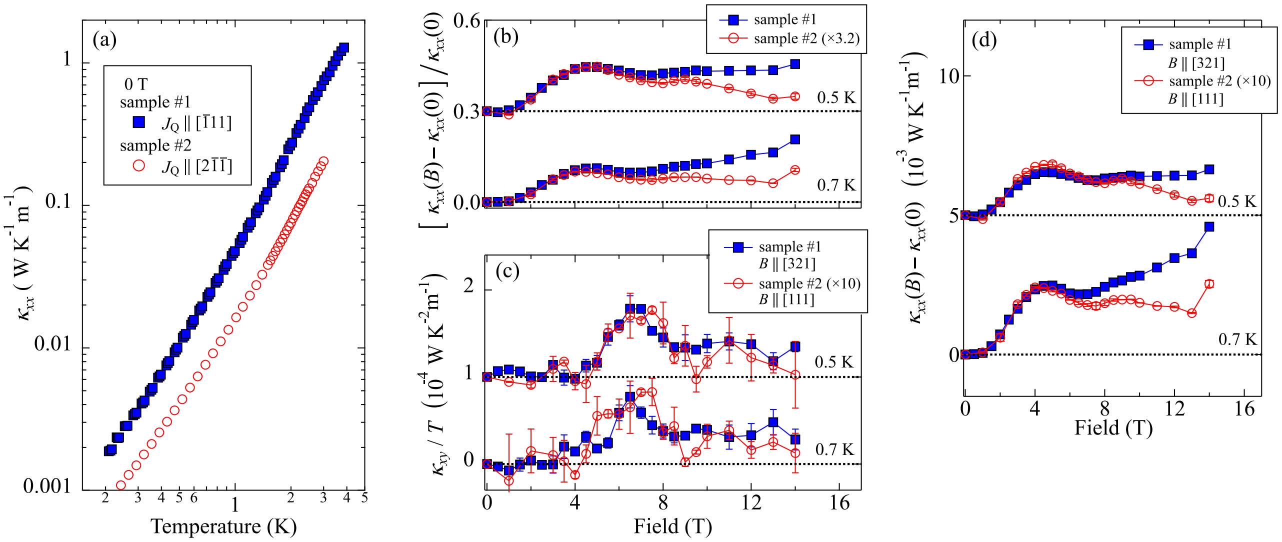

Supplementary Figure 6. (a) Temperature dependence of κ_{xx} for sample #1 and sample #2. Field B dependence of (b) normalized $(\kappa_{xx}(B) - \kappa_{xx}(0))/\kappa_{xx}(0)$, (c) κ_{xy}/T, and (d) unnormalized $\kappa_{xx}(B) - \kappa_{xx}(0)$ at $T = 0.5$ K and 0.7 K obtained in the field up and down processes for the two samples. The error bars are the maximum deviations of the data from the averages. Panels (c) and (d) are the components induced by the field and have the common scaling factor ~ 10 about the two samples.

all tendency of our data that the sample #1 has larger values of κ_{xx} and κ_{xy} than sample #2.

We finally discuss how the direction of the applied field may influence our result. For $\Delta\kappa_{xx}(B)$, the peak at around 4 T and the dips at 7 T are the anomalies that take place at the magnetic phase transition points reported previously. For fields higher than 9 T, that of sample #2 shows strong suppression in further increasing B. This is in sharp contrast to the case of sample #1. In a high-field phase for $\boldsymbol{B} \parallel [111]$ the multi-domain structure each consisting of single-$\boldsymbol{Q}$, having six equivalent(different) $\boldsymbol{Q}$-vectors is reported[9]. If the magnetic field is tilted from [111], the six domains are no longer equivalent and the domain distribution change, namely the average magnetic structure will change. Under such effect, is natural to have factor 10 difference common to $\Delta\kappa_{xx}(B)$ and $\kappa_{xy}(B)$ between two samples under different field angles.

We thus expect that the magnetic phases show the same dependence on the field $\boldsymbol{B} \parallel [321]$ and $\boldsymbol{B} \parallel [111]$ below 9 T, as has been confirmed in Supplementary Figs. 6(c) and S6(d). It has been shown previously that the AFM-SkL phase appears not only in the particular field direction $\boldsymbol{B} \parallel [111]$ but also along $\boldsymbol{B} \parallel [100]$ and $\boldsymbol{B} \parallel [110]$, although the shapes of the phase boundaries differ[12]. Therefore, we can safely conclude that the results we obtained for $\boldsymbol{B} \parallel [321]$ for sample #1 is the one that detects the AFM-SkL phase reported previously.

II. SUPPLEMENTARY NOTE 2: THEORETICAL

A. Details of the spin wave theory

We consider the spin Hamiltonian on a diamond lattice in Eq.(1) in the main text. The vectors $\boldsymbol{\delta}_1, \boldsymbol{\delta}_2$ and $\boldsymbol{\delta}_3$ showing uniaxial spin anisotropy along the bond directions of the lattice is

$$\begin{aligned}
4\boldsymbol{\delta}_1 =\ & (1,1,1)\,,(-1,1,1)\,,(1,-1,1)\,,(1,1,-1)\,,\\
& (-1,-1,1)\,,(1,-1,-1)\,,(-1,1,-1)\,,(-1,-1,-1)\,,\\
2\boldsymbol{\delta}_2 =\ & (1,0,1)\,,(0,1,1)\,,(-1,0,1)\,,(0,-1,1)\,,\\
& (1,1,0)\,,(1,-1,0)\,,(-1,1,0)\,,(-1,-1,0)\,,\\
& (1,0,-1)\,,(0,1,-1)\,,(-1,0,-1)\,,(0,-1,-1)\,,\\
4\boldsymbol{\delta}_3 =\ & (3,1,1)\,,(-3,1,1)\,,(3,-1,1)\,,(3,1,-1)\,,\\
& (-3,-1,1)\,,(3,-1,-1)\,,(-3,1,-1)\,,(-3,-1,-1)\,,\\
& (1,3,1)\,,(-1,3,1)\,,(1,-3,1)\,,(1,3,-1)\,,\\
& (-1,-3,1)\,,(1,-3,-1)\,,(-1,3,-1)\,,(-1,-3,-1)\,,\\
& (1,1,3)\,,(-1,1,3)\,,(1,-1,3)\,,(1,1,-3)\,,\\
& (-1,-1,3)\,,(1,-1,-3)\,,(-1,1,-3)\,,(-1,-1,-3)\,,
\end{aligned} \tag{3}$$

where we need to remove the ones not on the diamond lattice when implementing them into Eq.(1). In the following, we show the classical magnetic structure $\boldsymbol{m}_{\boldsymbol{r}}$ using the propagation vector $\boldsymbol{q}$ or $\boldsymbol{q}_m$, unit cell vector $\{\boldsymbol{a}_i\}$ and reciprocal lattice vectors $\{\boldsymbol{b}_i\}$ of the corresponding magnetic orderings. The propagation vectors can be more than one and take the values within the multiples of $\{\boldsymbol{b}_i\}$. Throughout this work, the coordinates of the vectors are

all commonly defined based on the original lattice (before the magnetic ordering in a unit of cell spacings), and so as the reciprocal ones. To be precise, the vectors are indexed in the unit cell of the paramagnetic crystal lattice of $MnSc_2S_4$ (space group 227, Fd-3m, lattice parameter $a = 10.606\mathring{A}$) and wave vectors are indexed in units of the corresponding reciptocal lattice.

Helical and fan phases.
The normalized vector spins of the classical helical and fan ground states are given by

$$\boldsymbol{m_r} \propto -\sin(\boldsymbol{q}\cdot\boldsymbol{r})\boldsymbol{e}_{1\bar{1}0} - \cos(\boldsymbol{q}\cdot\boldsymbol{r}+\phi)\boldsymbol{e}_{110} + M\boldsymbol{e_B}, \quad (4)$$

where $\phi = -\pi$ for the helical phase and $\phi = -3\pi/2$ for the fan phase, $\boldsymbol{e}_{\rm abc}$ is the unit vector along the [abc] direction, $\boldsymbol{e_B}$ is the unit vector along the magnetic field $\boldsymbol{B}$, and $\boldsymbol{q} = \frac{3\pi}{2}(1,1,0)$. The primitive translation vectors are $\boldsymbol{a}_1 = (2,2,0), \boldsymbol{a}_2 = \left(\frac{1}{2},-\frac{1}{2},0\right), \boldsymbol{a}_3 = (0,0,1)$, where there are 16 sites in the magnetic unit cell. The reciprocal lattice vectors are $\boldsymbol{b}_1 = \frac{\pi}{2}(1,1,0), \boldsymbol{b}_2 = 2\pi(1,-1,0), \boldsymbol{b}_3 = 2\pi(0,0,1)$.

Antiferromagnetic skyrmion lattice phase.
The normalized vector spins of the classical AFM-SkL ground state is given by

$$\boldsymbol{m_r} \propto \sum_{m=1}^{3}\left(\sin(\boldsymbol{q}_m\cdot\boldsymbol{r})\boldsymbol{e}_m - \cos(\boldsymbol{q}_m\cdot\boldsymbol{r} - 9\pi/8)\boldsymbol{e}_{111}\right) + M\boldsymbol{e_B}, \quad (5)$$

where $\boldsymbol{e}_l = \boldsymbol{e}_{\bar{1}\bar{1}2},\ \boldsymbol{e}_{1\bar{2}1},\ \boldsymbol{e}_{2\bar{1}\bar{1}}$, and $\boldsymbol{q}_1 = \frac{3\pi}{2}(1,-1,0),\ \boldsymbol{q}_2 = \frac{3\pi}{2}(1,0,-1),\ \boldsymbol{q}_3 = \frac{3\pi}{2}(0,1,-1)$. There are 384 sites in the magnetic unit cell spanned by $\boldsymbol{a}_1 = (4,0,-4),\ \boldsymbol{a}_2 = (0,4,-4),\ \boldsymbol{a}_3 = (1,1,1)$. The reciprocal lattice vectors are given by $\boldsymbol{b}_1 = \frac{\pi}{6}(2,-1,-1),\ \boldsymbol{b}_2 = \frac{\pi}{6}(-1,2,-1),\ \boldsymbol{b}_3 = \frac{2\pi}{3}(1,1,1)$.

Spin wave Hamiltonian.
For the spin wave analysis, we first introduce the three-dimensional rotation matrix as

$$R_{\boldsymbol{r}}^{\mu\nu} = n_{\boldsymbol{r}}^{\mu}n_{\boldsymbol{r}}^{\nu} + (\delta^{\mu\nu} - n_{\boldsymbol{r}}^{\mu}n_{\boldsymbol{r}}^{\nu})\cos\phi_{\boldsymbol{r}} - \sin\phi_{\boldsymbol{r}}\sum_{\rho}\epsilon^{\mu\nu\rho}n_{\boldsymbol{r}}^{\rho}, \quad (6)$$

where vector $\boldsymbol{n_r}$ and angle $\phi_{\boldsymbol{r}}$ are defined as

$$\boldsymbol{n_r} = \frac{\boldsymbol{m_r}\times\boldsymbol{e}^z}{|\boldsymbol{m_r}\times\boldsymbol{e}^z|}, \qquad \phi_{\boldsymbol{r}} = \arccos(\boldsymbol{e}^z\cdot\boldsymbol{m_r}). \quad (7)$$

This matrix satisfies $R_{\boldsymbol{r}}\boldsymbol{m_r} = \boldsymbol{e}^z$, meaning that it rotates the direction of the spin to the quantization axis (z-axis), which is the local gauge transformation changing the representation of the Hamiltonian but not the physical quantities.

We apply the Holstein-Primakoff transformation in the rotating frame,

$$R_{\boldsymbol{r}}\boldsymbol{S_r} \simeq \sqrt{\frac{S}{2}}(b_{\boldsymbol{r}} + b_{\boldsymbol{r}}^{\dagger})\boldsymbol{e}^x - i\sqrt{\frac{S}{2}}(b_{\boldsymbol{r}} - b_{\boldsymbol{r}}^{\dagger})\boldsymbol{e}^y + (S - b_{\boldsymbol{r}}^{\dagger}b_{\boldsymbol{r}})\boldsymbol{e}^z. \quad (8)$$

The spin Hamiltonian is approximated as $\hat{\mathcal{H}} \simeq E_{\rm cl.} + \hat{\mathcal{H}}_{\rm mag.}$, where the magnon Hamiltonian $\hat{\mathcal{H}}_{\rm mag.}$ is calculated as

$$\hat{\mathcal{H}}_{\rm mag.} = \sum_{\boldsymbol{r}}\varepsilon_{\boldsymbol{r}}b_{\boldsymbol{r}}^{\dagger}b_{\boldsymbol{r}} + \frac{1}{2}\sum_{\boldsymbol{r}}\left(\lambda_{\boldsymbol{r}}(b_{\boldsymbol{r}}^{\dagger})^2 + \text{h.c.}\right) + \frac{1}{2}\sum_{\boldsymbol{r},m,\boldsymbol{\delta}_m}\left(t_{\boldsymbol{r},\boldsymbol{\delta}_m}b_{\boldsymbol{r}}^{\dagger}b_{\boldsymbol{r}+\boldsymbol{\delta}_m} + \Delta_{\boldsymbol{r},\boldsymbol{\delta}_m}b_{\boldsymbol{r}}^{\dagger}b_{\boldsymbol{r}+\boldsymbol{\delta}_m}^{\dagger} + \text{H.c.}\right), \quad (9)$$

where $\varepsilon_{\boldsymbol{r}}$, $\lambda_{\boldsymbol{r}}$, $t_{\boldsymbol{r},\boldsymbol{\delta}_m}$, and $\Delta_{\boldsymbol{r},\boldsymbol{\delta}_m}$ are determined by the parameters in the original spin Hamiltonian.

We perform Fourier transformation $b_{\boldsymbol{r}} = \sqrt{N_s}^{-1}\sum_{\boldsymbol{k}}b_{\boldsymbol{k},\alpha}e^{i\boldsymbol{k}\cdot\boldsymbol{r}}$, where $N_s = 16$ and 384 for helical/fan and AFM-SkL phases, and $\alpha = 1,2,\cdots,N_s$ is the sublattice site index within the magnetic unit cell.

The magnon Hamiltonian is rewritten using $\Phi_{\boldsymbol{k}} = (b_{\boldsymbol{k},1},\cdots,b_{\boldsymbol{k},N_s})$ as

$$\mathcal{H}_{\rm mag.} = \frac{1}{2}\sum_{\boldsymbol{k}}\Phi_{\boldsymbol{k}}^{\dagger}H_{\rm BdG}(\boldsymbol{k})\Phi_{\boldsymbol{k}} + \text{const.}, \quad (10)$$

$$H_{\rm BdG}(\boldsymbol{k}) = \begin{pmatrix}\Xi(\boldsymbol{k}) & \Delta(\boldsymbol{k})\\ \Delta^*(-\boldsymbol{k}) & \Xi^*(-\boldsymbol{k})\end{pmatrix}, \quad (11)$$

where $\Xi(\boldsymbol{k})$ and $\Delta(\boldsymbol{k})$ are $N_s\times N_s$ matrices consisting of parameters in Supplementary Eq. (9).

For practical purpose, we add a tiny constant $\sim O(0.01)$ to $\varepsilon_{\boldsymbol{r}}$ in Supplementary Eq.(9) by hand, which is the diagonal part of $H_{\rm BdG}(\boldsymbol{k})$, in order to guarantee the stabilization of the assumed spin configurations in Supplementary Eqs.(4) and (5). In diagonalizaing the Hamiltonian, this constant does not change the period of the magnetic structure (size of the unit cell) of the ground state, labeled by up to three wave numbers in $\boldsymbol{m_r}$, but excludes the higher order harmonics that may slightly alter the magnetic moments inside the unit cell. However, its order is of that tiny constant $O(0.01)$, and even if the higher energy part of the magnon band may slightly change at this order, the redistribution of Berry curvature and its influence on the thermal Hall coefficients is minor because of the Bose statistics. For this reason, the tiny constant is taken as field-independent and does not change the outcome. This treatment is legitimated as good approximation, because the Monte Carlo simulation[9] guarantees that even in a substantially large magnetic field in the AFM-SkL phase, the ground state given in Supplementary Eqs.(4) and (5) is well stabilized apart from a small corrections we have discarded. This treatment supports the positive-definite spectrum of the magnon bands[8] of Supplementary Eqs.(4) and (5) and

largely simplifies the process of dealing with a complex magnetic structure.

The magnon bands and eigenstates can be obtained by solving the eigenvalue equations, $\Sigma^z H_{\rm BdG}(\boldsymbol{k})\boldsymbol{t}_n(\boldsymbol{k}) = \varepsilon_n(\boldsymbol{k})\boldsymbol{t}_n(\boldsymbol{k})$, with $\Sigma^z = \sigma^z \otimes I_{N_s\times N_s}$. By using magnon eigenstates, $\boldsymbol{t}_n(\boldsymbol{k})$, we obtain the Berry curvature and the thermal Hall conductivity in the main text.

B. Variation of magnon bands

In Supplementary Fig. 7(a) we show the magnon bands obtained at $B = 2\,\mathrm{T}$ by artificially setting $J_\parallel = 0$. There, we find that although the band structures look similar to the one shown in Fig. 2(a) in the main text, they have mostly $\Omega^n_{xy} = 0$, indicating that the helical spin configuration does not have room to exhibit a thermal Hall effect. This indicates that the anisotropic magnetic exchange interactions coming from the spin-orbit coupling are essential to have a finite Berry curvature.

Supplementary Figure 7(b) shows the magnon bands for AFM-SkL phase in higher fields. At 5 T, we find a much larger gap compared to the case of 4 T, while for 6 and 6.5 T the gap decreases. At the same time, we find tens of magnon bands in the energy window of $\varepsilon \lesssim 2\,\mathrm{K}$, while its number seems to decrease with increasing the field. This will influence the number of magnon carriers thermally excited, and explain the continuous decrease of κ_{xx} in the experiment toward 8 T.

For κ_{xy}/T, not only the number of low magnon bands but the sign of Ω^n_{xy} matters, which cannot be visibly understood solely by the present plot, and will be examined in the next subsection.

C. Details about the contributions from magnon bands to κ_{xy}

We now examine how the variation of magnon bands influences κ_{xy} by examining their contribution in detail. The Berry curvature $\Omega^{(n)}_{\mu\nu}(\boldsymbol{k})$ ($n = 1, 2, \cdots, N_s$) is described as

$$\begin{aligned}\Omega^{(n)}_{\mu\nu}(\boldsymbol{k}) &= -2\mathrm{Im}\left[\frac{\partial \boldsymbol{t}^\dagger_n(\boldsymbol{k})}{\partial k_\mu}\Sigma^z\frac{\partial \boldsymbol{t}_n(\boldsymbol{k})}{\partial k_\nu}\right] \\ &= -2\sum_{m=1}^{2N_s}(1-\delta_{m,n})(\Sigma^z)_{m,m} \\ &\quad \frac{\mathrm{Im}\left[\boldsymbol{t}^\dagger_n(\boldsymbol{k})\frac{\partial H_{\rm BdG}(\boldsymbol{k})}{\partial k_\mu}\boldsymbol{t}_m(\boldsymbol{k})\boldsymbol{t}^\dagger_m(\boldsymbol{k})\frac{\partial H_{\rm BdG}(\boldsymbol{k})}{\partial k_\nu}\boldsymbol{t}_n(\boldsymbol{k})\right]}{(\varepsilon_n(\boldsymbol{k})-\varepsilon_m(\boldsymbol{k}))^2+\delta},\end{aligned} \tag{12}$$

where $m = N_s + 1, \cdots, 2N_s$ denotes the particle-hole pairs. In the actual calculation, the wave numbers k_ν, k_μ runs over k_x, k_y, which corresponds to the real space direction $e_x = [1\bar{1}\bar{1}]$, $e_y = [\bar{1}4\bar{5}]$ to conform to the experiment, as given in the inset of Fig. 2(e) in the main text. In principle, one can take different directions of k_x and k_y at their choice. The first line in Supplementary Eq.(12) gives the expressions using only the information about the n-th band, while the second expression includes the inter-band matrix elements over the whole bands, making use of the completeness of the basis. We use the latter for numerical evaluation, where we introduce an infinitesimal positive number δ to reduce the numerical error due to divergence and take $\delta \to 0$.

We now test how the number of bands included in the calculation of κ_{xy}, denoted as N'_s would influence the results. After obtaining the distribution of $\Omega^{(n)}_{xy}(\boldsymbol{k})$ over all the magnon bands, we confine the summation to the lowest N'_s-bands in the following formula,

$$\kappa_{xy} = -\frac{k_{\rm B}^2 T}{\hbar}\int_{\rm BZ}\frac{d^3\boldsymbol{k}}{(2\pi)^3}\sum_{n=1}^{N'_s} c_2[f(\varepsilon_n(\boldsymbol{k}))]\Omega^{(n)}_{xy}(\boldsymbol{k}). \tag{13}$$

Supplementary Figure 8(a) shows κ_{xy} as function of $\boldsymbol{B} \parallel [111]$ for different choices of $N'_s = 8, 16, 24$ and 384(full) at $T = 0.2$ and $0.5\,\mathrm{K}$. At $T = 0.2\,\mathrm{K}$, the results converge already at $N'_s = 16$, showing that only 10 bands or so contribute to κ_{xy}. However, at $T = 0.5\,\mathrm{K}$, the results differ much even when we increase as large as $N'_s = 24$. The same tendency holds for $\boldsymbol{B} \parallel [321]$ shown in Supplementary Fig. 8(b). For clarification, we plot in Supplementary Fig. 8(c) the lowest N'_s-bands with $N'_s = 8, 16, 24$. One finds that already at $T = 0.5\,\mathrm{K}$, the magnon excitation following Bose statistics easily exceeds the energy window $\sim 2\,\mathrm{K}$ that accomodates $N'_s = 24$. We thus clarify that in the AFM-SkL phase, the characteristic dense magnon bands contribute up to high energies in the thermal Hall effect.

D. Real-space spin texture and U(1) gauge fields in AFM-SkX on the triangular lattice

Here, we give the details of the calculations performed to obtain Fig. 4(e) in the main text. We consider the two-dimensional xy-plane that describes a single [111]-layer of $MnSc_2S_4$ that form the triangular lattice by taking [111] as z-axis. The orientations of the ordered magnetic moments of the AFM-SkL (see Fig. 1(b) and Fig. 4(e) in the main text) is given by the unit vector $\boldsymbol{m}(\boldsymbol{r})$ whose elements are given in the Cartesian coordinate as

$$\begin{aligned}\boldsymbol{m}(\boldsymbol{r}) =& A^{-1}\left[\sum_{m=1}^{3}\left(-\sin(\boldsymbol{q}_m\cdot\boldsymbol{r})\boldsymbol{e}^m \right.\right.\\ &\left.\left.+\cos\left(\boldsymbol{q}_m\cdot\boldsymbol{r}-\frac{9\pi}{8}\right)\boldsymbol{e}^z\right)+M\boldsymbol{e}^z\right],\end{aligned} \tag{14}$$

where A is a normalization factor to keep $|\boldsymbol{m}(\boldsymbol{r})| = 1$, M is the spatially uniform magnetization due to a finite magnetic field, and $\boldsymbol{e}^m$ ($m = 1, 2, 3$) are the unit vectors on the three-dimensional plane pointing in the direction

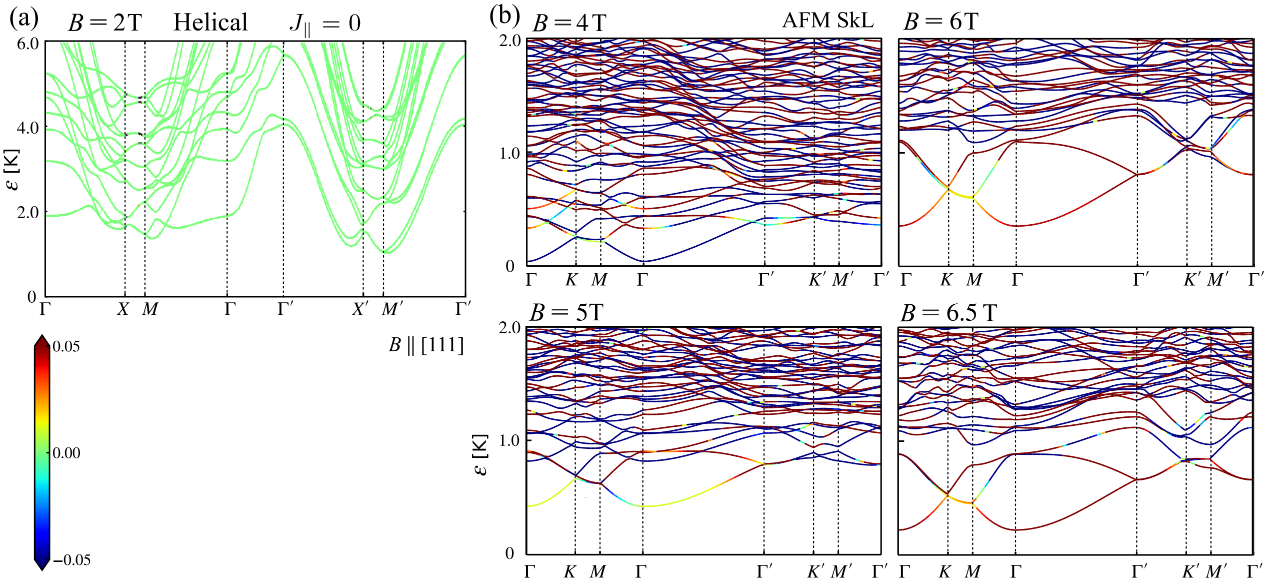

Supplementary Figure 7. Magnon bands obtained by the spin-wave theory in a field $\boldsymbol{B} \parallel [111]$. (a) Helical state with $J_{\parallel} = 0$ at $B = 2\,\mathrm{T}$ to be compared with the the one with $J_{\parallel} \neq 0$ in Fig. 2(a) of the main text, both with $N_s = 16$. (b-d) AFM-SkL state with $N_s = 384$ at $B = 4, 5, 6, 6.5\,\mathrm{T}$. Magnon bands are shown in the color density plot of the Berry curvature $\Omega_{xy}^{(n)}$. The case of $\Omega_{xy}^{(n)} > 0.05$ and < -0.05 are all plotted in red and blue, respectively.

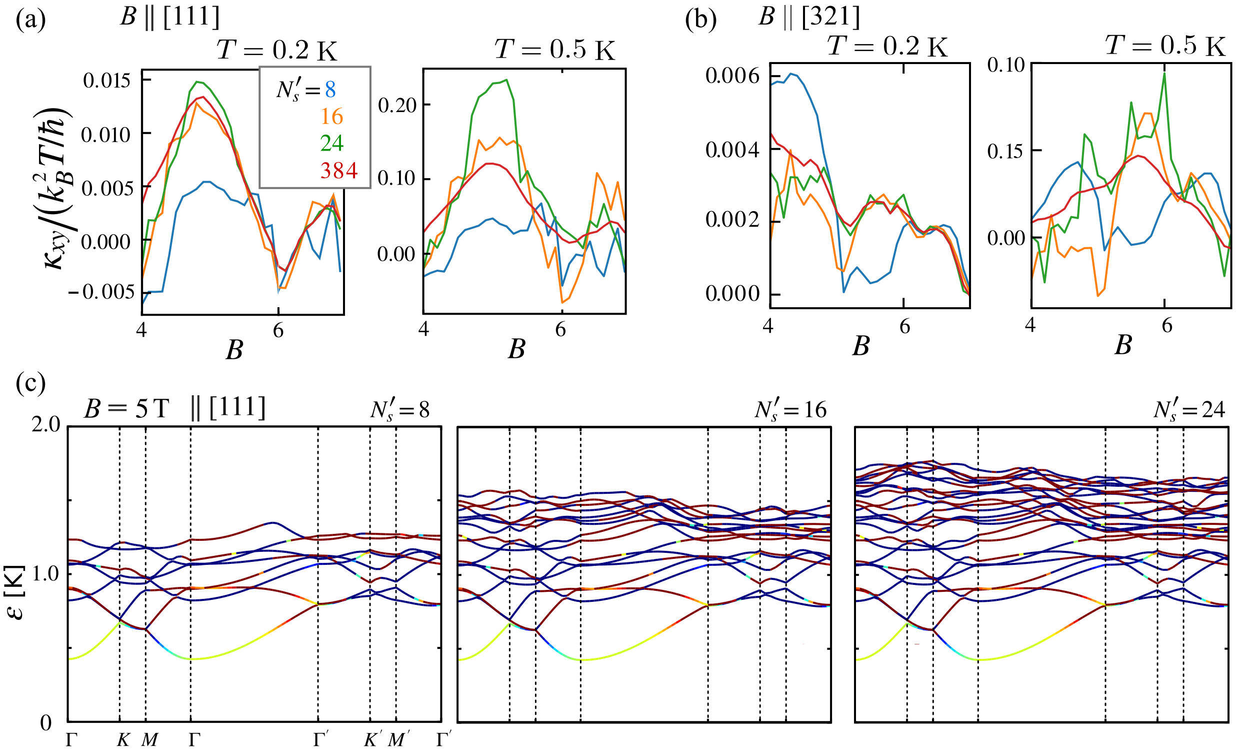

Supplementary Figure 8. (a,b) $\kappa_{xy}/(k_{\mathrm{B}}^2 T/\hbar)$ of the AFM-SkL phase obtained using the lowest N_s'-bands ($N_s' = 8, 16, 24, 384$) in taking the integral in for the field directions $\boldsymbol{B} \parallel [111]$ and [321]. (c) The description of lowest N_s' bands ($N_s' = 8, 16, 24$) at $B = 5\,\mathrm{T}$ and $\boldsymbol{B} \parallel [111]$.

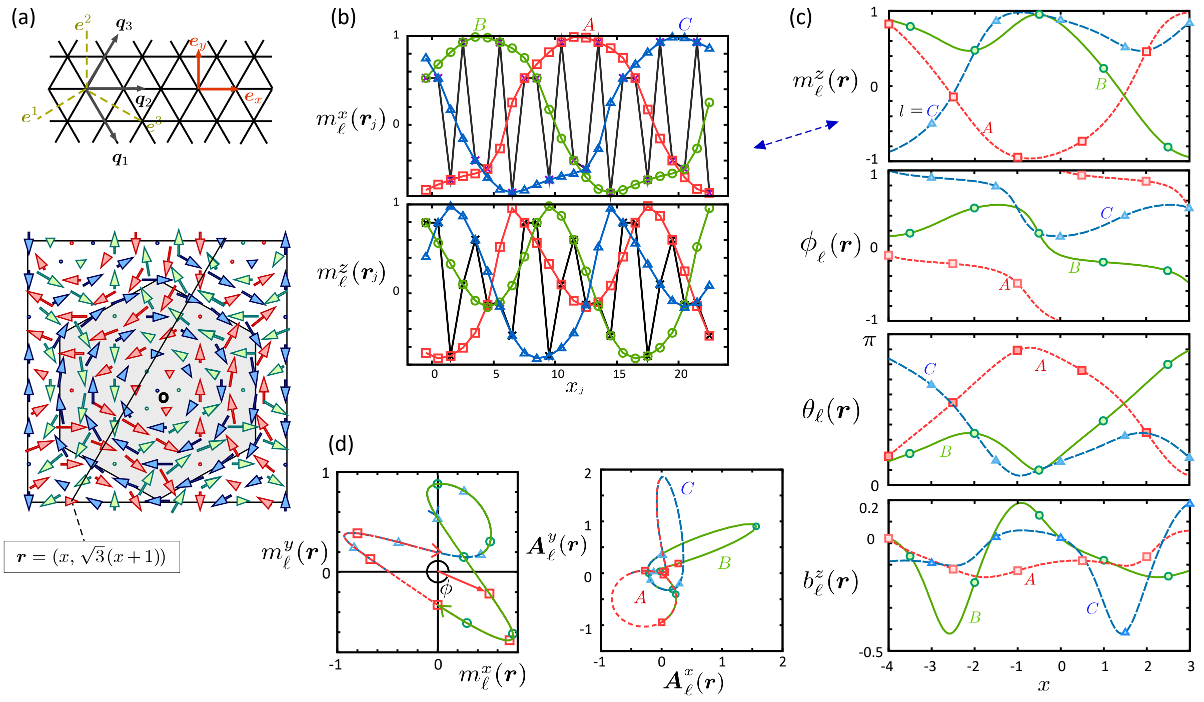

Supplementary Figure 9. (a) Vectors defined in the two-dimensional xy-plane used in the formulation. (b) Magnetic structrues $m^x_\ell(\boldsymbol{r}_j)$ and $m^z_\ell(\boldsymbol{r}_j)$ obtained for discrete lattice sites $\boldsymbol{r}_j = (x_j, 3\sqrt{3}/2)$. The one using Supplementary Eq.(14) give the magnetization for all three sublattices, whereas those in Supplementary Eq.(18) give $\boldsymbol{m}_\ell$ of $\ell = A, B$, and C sublattices separately, showing that the two equations are consistent. (c) The magnetization $m^z_\ell(\boldsymbol{r})$, $\phi_\ell(\boldsymbol{r})$, $\theta_\ell(\boldsymbol{r})$, and $b^z_\ell(\boldsymbol{r})$ along the $\boldsymbol{r} = (x, \sqrt{3}(x+1))$ line shown in the lower-panel of (a), whose tangent give the angle of sublattice sites. The data points are the values for the discrete lattice points, and the lines are the obtained as continuous functions of $\boldsymbol{r}$. (d) Along the same line, the variation of magnetization on the plane of $m^x_\ell(\boldsymbol{r})$ versus $m^y_\ell(\boldsymbol{r})$. and the vector potential on the plane of $A^x_\ell(\boldsymbol{r})$ and $A^y_\ell(\boldsymbol{r})$ are shown.

perpendicular to $\boldsymbol{q}_m$ as

$$\boldsymbol{e}^1 = -\frac{\sqrt{3}\boldsymbol{e}^x + \boldsymbol{e}^y}{2}, \;\; \boldsymbol{e}^2 = -\boldsymbol{e}^y, \;\; \boldsymbol{e}^3 = \frac{\sqrt{3}\boldsymbol{e}^x - \boldsymbol{e}^y}{2}, \quad (15)$$

$$\boldsymbol{q}_1 = \frac{3\pi}{2}\frac{\boldsymbol{e}_x - \sqrt{3}\boldsymbol{e}_y}{2}, \;\; \boldsymbol{q}_2 = \frac{3\pi}{2}\boldsymbol{e}_x, \;\; \boldsymbol{q}_3 = \frac{3\pi}{2}\frac{\boldsymbol{e}_x + \sqrt{3}\boldsymbol{e}_y}{2}, \quad (16)$$

where we regard $\boldsymbol{e}^\mu$ and $\boldsymbol{e}_\mu$ as those defined in the spin space and in real space, respectively (see Supplementary Fig. 9(a)).

Let us consider the spatial variation of magnetic moments separately for the three sublattices $\ell = A, B, C$ to construct the U(1) gauge fields. This can be done by using the same $\boldsymbol{e}^m$ ($m = 1, 2, 3$) as those given in Supplementary Eq.(15) but by taking different periods of propagation vectors

$$\boldsymbol{Q}_1 = \frac{\pi}{6}\frac{\boldsymbol{e}_x - \sqrt{3}\boldsymbol{e}_y}{2}, \;\; \boldsymbol{Q}_2 = \frac{\pi}{6}\boldsymbol{e}_x, \;\; \boldsymbol{Q}_3 = \frac{\pi}{6}\frac{\boldsymbol{e}_x + \sqrt{3}\boldsymbol{e}_y}{2}, \quad (17)$$

and by shifting the origin depending on the sublattices to $\tilde{\boldsymbol{r}}_\ell = \boldsymbol{r} - \boldsymbol{r}^\ell_0$ with $\boldsymbol{r}^A_0 = 0$, $\boldsymbol{r}^B_0 = (16, 0)$, $\boldsymbol{r}^C_0 = (8, 0)$. Then we find the magnetic moment on sublattice ℓ as

$$\boldsymbol{m}_\ell(\boldsymbol{r}) = A^{-1}\left[\sum_{m=1}^{3}\Big(-\sin(\boldsymbol{Q}_m \cdot \tilde{\boldsymbol{r}}_\ell)\boldsymbol{e}^m + \cos\left(\boldsymbol{Q}_m \cdot \tilde{\boldsymbol{r}}_\ell - \frac{9\pi}{8}\right)\boldsymbol{e}^z\Big) + M\boldsymbol{e}^z\right]. \quad (18)$$

Since $\boldsymbol{m}_\ell(\boldsymbol{r})$ is described using $(\theta_\ell(\boldsymbol{r}), \phi_\ell(\boldsymbol{r}))$, we can rewrite these elements as

$$\sin\theta_\ell(\boldsymbol{r})\cos\phi_\ell(\boldsymbol{r}) = \frac{\sqrt{3}}{2A}\Big(\sin(\boldsymbol{Q}_1\cdot\tilde{\boldsymbol{r}}_\ell) - \sin(\boldsymbol{Q}_3\cdot\tilde{\boldsymbol{r}}_\ell)\Big),$$
$$\sin\theta_\ell(\boldsymbol{r})\sin\phi_\ell(\boldsymbol{r}) = \frac{-1}{2A}\Big(\sin(\boldsymbol{Q}_1\cdot\tilde{\boldsymbol{r}}_\ell) + 2\sin(\boldsymbol{Q}_2\cdot\tilde{\boldsymbol{r}}_\ell) + \sin(\boldsymbol{Q}_3\cdot\tilde{\boldsymbol{r}}_\ell)\Big),$$
$$\cos\theta_\ell(\boldsymbol{r}) = \frac{1}{A}\Big(\sum_m \cos\left(\boldsymbol{Q}_m\cdot\tilde{\boldsymbol{r}}_\ell - \frac{9\pi}{8}\right) + M\Big), \quad (19)$$

which gives the description of magnetic moments in the continuous space $\boldsymbol{r}$ in the xy plane. Supplementary Figure 9(b) shows the comparison of $\boldsymbol{m}(\boldsymbol{r})$ in Supplementary Eq.(14) and $\boldsymbol{m}_\ell(\boldsymbol{r})$ in Supplementary Eq.(18) along

the $\boldsymbol{r}_j = (x_j, 0)$ line calculated for discrete lattice points where we set $M = 0$. At each lattice point, they cross while the spatial periods for the continuous $\boldsymbol{r}$ differ. This shows that the two equations are consistent with each other.

The U(1) gauge field is constructed using slowly varying sets of $\boldsymbol{m}_\ell(\boldsymbol{r})$. The vector potential for the three sublattices is given as

$$\boldsymbol{A}_\ell(\boldsymbol{r}) = -\frac{\cos\phi_\ell(\boldsymbol{r})}{\tan\theta_\ell(\boldsymbol{r})}\nabla \boldsymbol{m}_\ell^y(\boldsymbol{r}) + \frac{\sin\phi_\ell(\boldsymbol{r})}{\tan\theta_\ell(\boldsymbol{r})}\nabla \boldsymbol{m}_\ell^x(\boldsymbol{r}), \quad (20)$$

and the fictitious magnetic field generated as a rotation of the vector field $\boldsymbol{A}_\ell(\boldsymbol{r})$ is given as

$$\begin{aligned} b_\ell^z(\boldsymbol{r}) &= \partial_x A_\ell^y(\boldsymbol{r}) - \partial_y A_\ell^x(\boldsymbol{r}) \\ &= \boldsymbol{m}_\ell(\boldsymbol{r}) \cdot \big(\partial_x \boldsymbol{m}_\ell(\boldsymbol{r}) \times \partial_y \boldsymbol{m}_\ell(\boldsymbol{r})\big), \end{aligned} \quad (21)$$

where

$$\begin{aligned} \partial_\mu \boldsymbol{m}_\ell^x(\boldsymbol{r}) =& \frac{\sqrt{3}}{2A}\Big((\boldsymbol{Q}_1)_\mu \cos(\boldsymbol{Q}_1 \cdot \tilde{\boldsymbol{r}}_\ell) - s(\boldsymbol{Q}_3)_\mu \cos(\boldsymbol{Q}_3 \cdot \tilde{\boldsymbol{r}}_\ell)\Big), \\ \partial_\mu \boldsymbol{m}_\ell^y(\boldsymbol{r}) =& \frac{1}{2A}\Big((\boldsymbol{Q}_1)_\mu \cos(\boldsymbol{Q}_1 \cdot \tilde{\boldsymbol{r}}_\ell) + 2(\boldsymbol{Q}_2)_\mu \cos(\boldsymbol{Q}_2 \cdot \tilde{\boldsymbol{r}}_\ell) \\ &+ (\boldsymbol{Q}_3)_\mu \cos(\boldsymbol{Q}_3 \cdot \tilde{\boldsymbol{r}}_\ell)\Big), \\ \partial_\mu \boldsymbol{m}_\ell^z(\boldsymbol{r}) =& -\frac{1}{A}\Big[\sum_m (\boldsymbol{Q}_m)_\mu \sin\big(\boldsymbol{Q}_m \cdot \tilde{\boldsymbol{r}}_\ell - \frac{9\pi}{8}\big)\Big]. \end{aligned} \quad (22)$$

Supplementary Figure 9 (c) shows $m_\ell^z(\boldsymbol{r})$, $\phi_\ell(\boldsymbol{r})$, $\theta_\ell(\boldsymbol{r})$, and $b_\ell^z(\boldsymbol{r})$ separately for the three sublattices. They are the continuous functions of $\boldsymbol{r}$ where we put the symbols at the discrete lattice points. The x, y components of magnetization and vector potential along the same line are shown in Supplementary Fig. 9(d). Based on these calculations, the vector field, $\boldsymbol{A}_\ell(\boldsymbol{r})$, and corresponding $b_\ell^z(\boldsymbol{r})$ are shown for the three sublattices in Fig. 4(e) in the main text.

* takeda.hikaru@issp.u-tokyo.ac.jp

† masataka.kawano@tum.de

[1] V. Fritsch, J. Hemberger, N. Büttgen, E.-W. Scheidt, H.-A. Krug von Nidda, A. Loidl, and V. Tsurkan, Phys. Rev. Lett. **92**, 116401 (2004).

[2] S. Gao, O. Zaharko, V. Tsurkan, Y. Su, J. White, G. Tucker, B. Roessli, F. Bourdarot, R. Sibille, D. Chernyshov, T. Fennell, A. Loidl, D.C., and C. Rüegg, Nature Phys. **13**, 157 (2017).

[3] W. Münzer, A. Neubauer, T. Adams, S. Mühlbauer, C. Franz, F. Jonietz, R. Georgii, P. Böni, B. Pedersen, M. Schmidt, A. Rosch, and C. Pfleiderer, Phys. Rev. B **81**, 041203 (2010).

[4] H. Oike, A. Kikkawa, N. Kanazawa, Y. Taguchi, M. Kawasaki, Y. Tokura, and F. Kagawa, Nature Physics **12**, 62 (2016).

[5] Y. Okamura, F. Kagawa, S. Seki, and Y. Tokura, Nature Communications **7**, 12669 (2016).

[6] A. Bauer, M. Garst, and C. Pfleiderer, Phys. Rev. B **93**, 235144 (2016).

[7] H. C. Chauhan, B. Kumar, and S. Ghosh, Scientific Reports **12**, 15971 (2022).

[8] M. Akazawa, H.-Y. Lee, H. Takeda, Y. Fujima, Y. Tokunaga, T.-h. Arima, J. H. Han, and M. Yamashita, Phys. Rev. Res. **4**, 043085 (2022).

[9] S. Gao, H. D. Rosales, F. A. G. Albarracin, G. K. Tsurkan, T. Fennell, P. Steffens, M. Boehm, P. Cermak, A. Schneldewind, E. Ressouche, D. Cabra, C. Rüegg, and O. Zaharko, Nature **586**, 37 (2020).

[10] M. Yamashita, M. Akazawa, M. Shimozawa, T. Shibauchi, Y. Matsuda, H. Ishikawa, T. Yajima, Z. Hiroi, M. Oda, H. Yoshida, H.-Y. Lee, J. H. Han, and N. Kawashima, Journal of Physics: Condensed Matter **32**, 074001 (2019).

[11] Y. Shiomi, Y. Onose, and Y. Tokura, Phys. Rev. B **81**, 054414 (2010).

[12] H. D. Rosales, F. A. G. Albarracín, K. Guratinder, V. Tsurkan, L. Prodan, E. Ressouche, and O. Zaharko, Phys. Rev. B **105**, 224402 (2022).
